# Supplementary material for: Modulating the activities of chloroplasts and mitochondria promotes adenosine triphosphate production and plant growth
Source: Quant Plant Biol. 2021 May 4;2:e7. doi: 10.1017/qpb.2021.7 (PMC10095973; doi:10.1017/qpb.2021.7)
Supplement: Supplementary file 1 [file qpbsup.zip › S2632882821000072sup001.pdf]

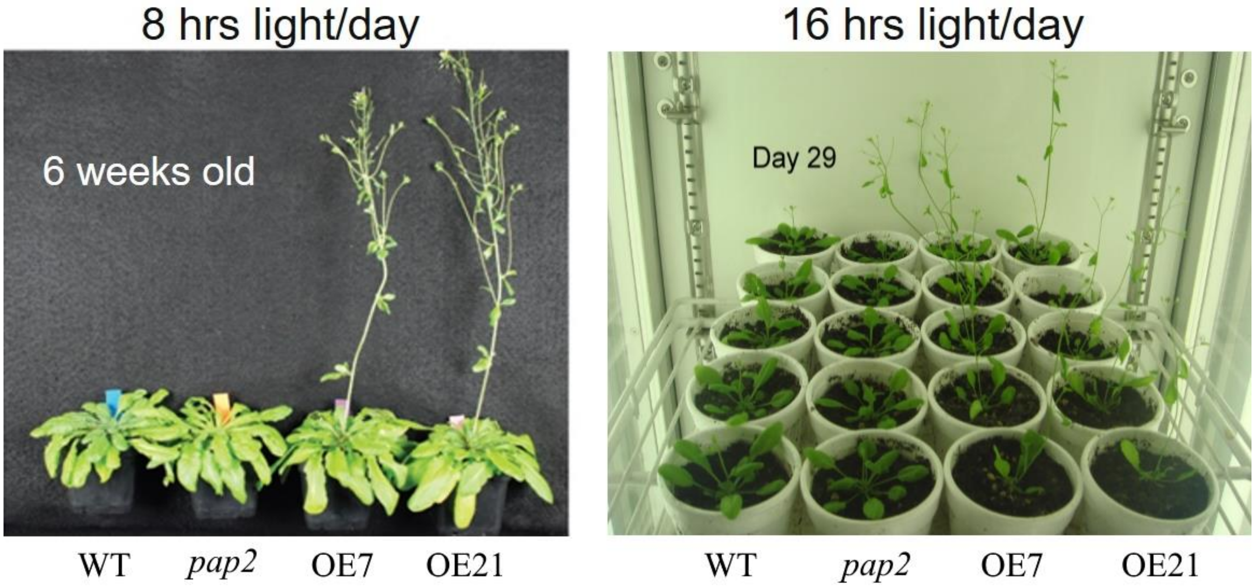

Supplementary Figure S1. The OE lines grew faster under both SD (8h/16h) and LD (16h/8h) conditions.

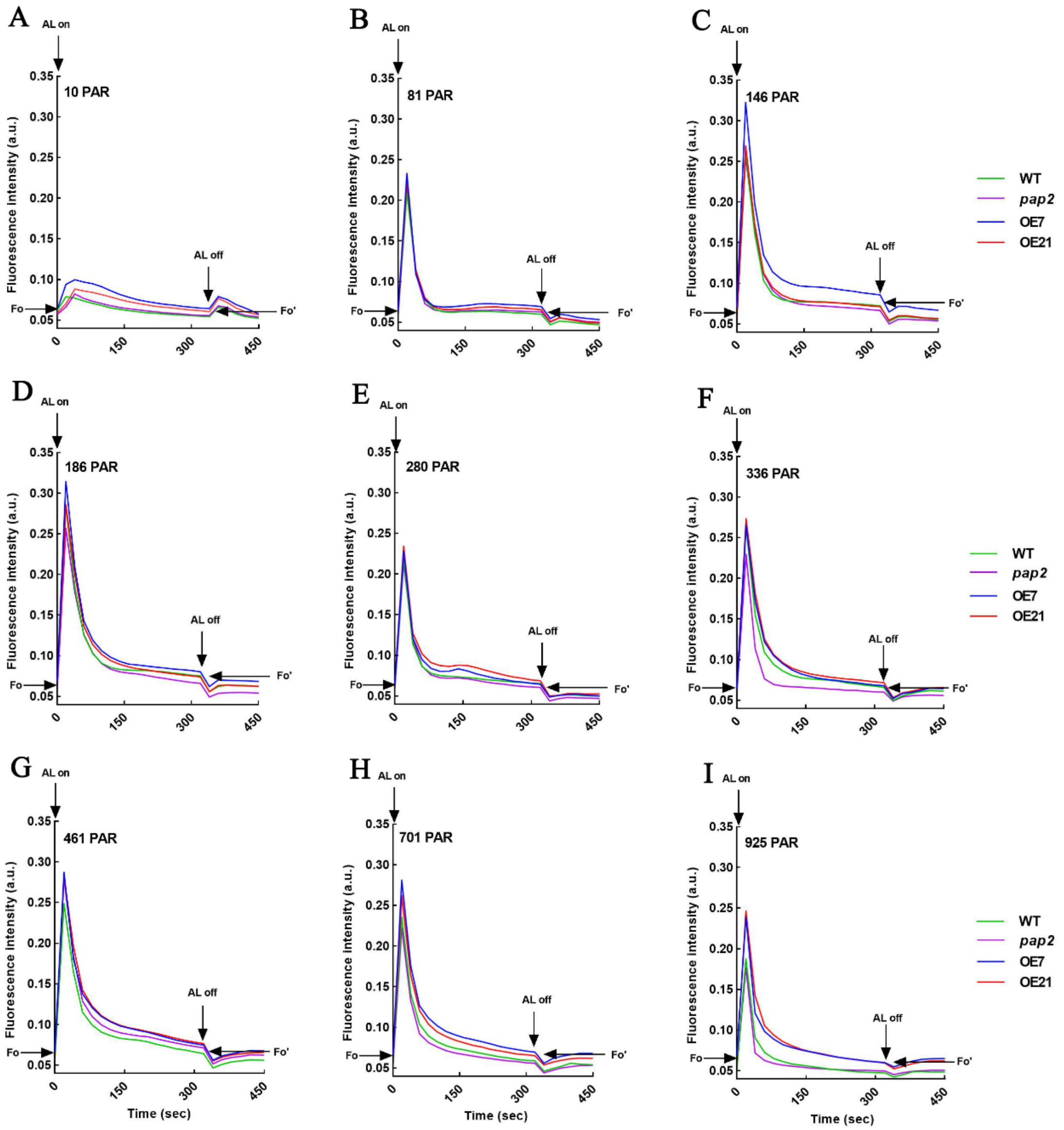

Supplementary Figure S2. Fluorescence kinetics of WT, *pap2*, OE7 and OE21 lines. 3-week-old plants were dark-acclimated for at least 1 hour before the measurement. Actinic light (AL) of 10, 81, 146, 186, 280, 336, 461, 701, 925 PAR was applied for 315 sec. Each data represents the mean of six biological replicates. **AL on**, actinic light on; **AL off**, actinic light off; **PAR**, photosynthetic active radiation (μmol photons $m^{-2}s^{-1}$); **Fo**, minimal fluorescence yield in the dark; **Fo'**, minimal fluorescence yield after illumination.

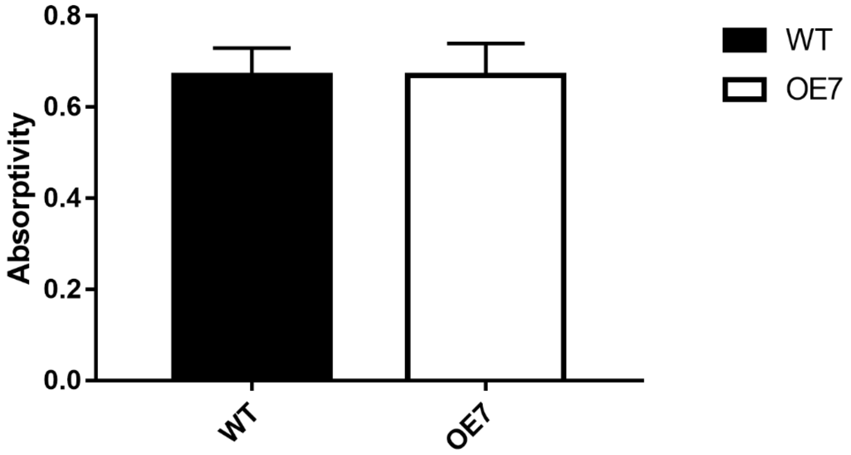

Supplementary Figure S3. Leaf absorptivity is similar between WT line and AtPAP2 overexpression line (OE7). Absorptivity of 20-day-old leaves were determined by IMAGING-PAM M-Series Maxi Version (WALZ, Germany). First an NIR (near-infrared)-remission image and then a R (red)-remission image was measured, the absorptivity was automatically calculated pixel by pixel according to the equation absorptivity = 1 - R/NIR. This approach is based on the empirical fact that pigments that contribute to the absorption of PAR (photosynthetic active radiation) do not show significant absorption bands in the NIR spectral region. On the other hands, pigments that absorb NIR are likely to also absorb Red light. Bar graph represents the mean value of absorptivity with SD (n = 63).

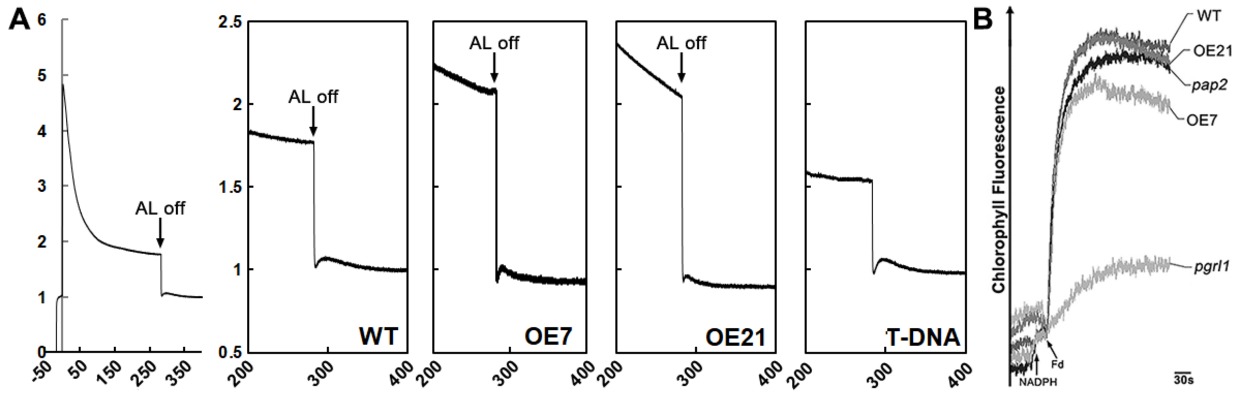

Supplementary Figure S4. ***In vivo*** **analysis of the cyclic electron flow rate of 20-day-old plants.** **A.** Post-illumination chlorophyll fluorescence transient for the measuring of NAD(P)H dehydrogenase (NDH)-CEF. Typical induction kinetics of chlorophyll fluorescence in a dark-acclimated leaf of WT Arabidopsis under illumination with actinic light (AL, 50 μmol photon m^{-2} s^{-1}) were shown. Changes in chlorophyll fluorescence after the cessation of the actinic illumination were compared at a higher time resolution. Chlorophyll fluorescence was normalized to the original level in the dark (F_0). **B.** Ruptured chloroplast assay for the quantification of antimycin A-sensitive Fd-dependent CEF. After the addition of NADPH and Fd to freshly ruptured chloroplasts, the increase in chlorophyll fluorescence was measured (n = 3). The *pgrl1* mutant served as a negative control.

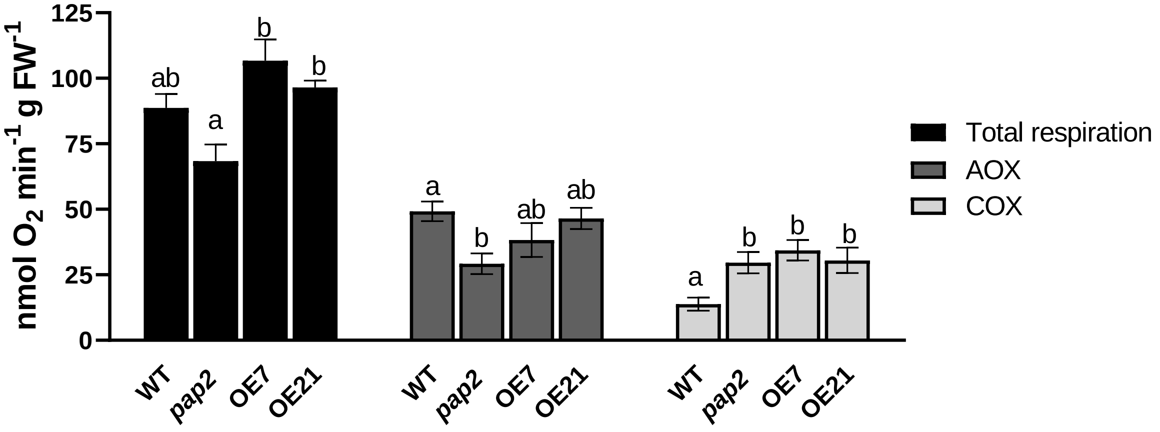

Supplementary Figure S5. Respiratory rate of 21-day-old leaves. Total respiration rate, AOX pathway respiration rate (the sensitivity of O_2 uptake to 10mM SHAM) and COX pathway respiration rate (the sensitivity of O_2 uptake to 4 mM KCN in the presence of 10mM SHAM) were expressed in nmol O_2 min^{-1} g FW^{-1} of WT, *pap2*, OE7 and OE21. Different letters indicate significant statistical differences analyzed by post-hoc Tukey's HSD test ($P < 0.05$) ($n = 5$; means ± SEM). **AOX**, alternative oxidase; **COX**, cytochrome c oxidase; **SHAM**, salicylhydroxamic acid; **KCN**, potassium cyanide.

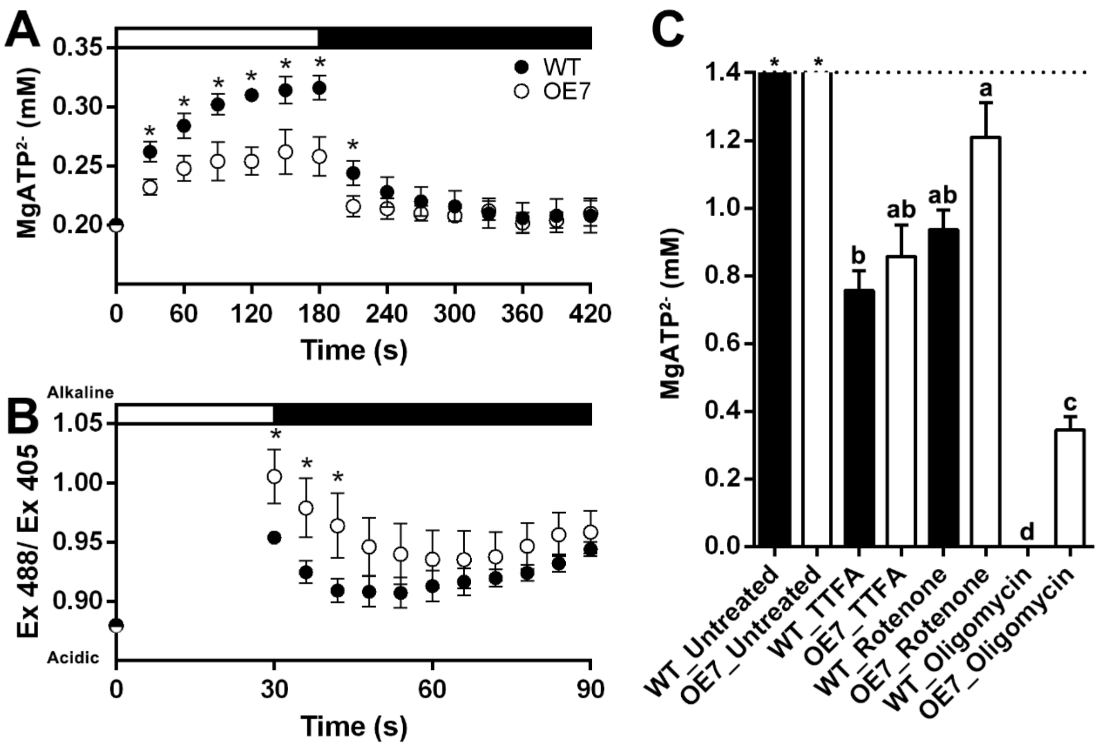

Supplementary Figure S6. Assessing organelle activities using fluorescent sensors. (a) Change in apparent $MgATP^{2-}$ concentration in the chloroplast stroma. 10-day-old WT and OE7 cotyledon expressing TKTP-AT1.03 were illuminated (296 µmol photon m^{-2} s^{-1}) for 3 mins, followed by 5 mins in the dark. (b) Changes in the mitochondrial matrix pH sensor ratio (Ex 488/ Ex 405). 10-day-old WT and OE7 cotyledon expressing matrix-localized pH sensor, mt-cpYFP were illuminated (296 µmol photon m^{-2} s^{-1}) for 30 s, followed by 1 min in the dark. Black and white bars indicate the dark and illumination periods, respectively. Average value of 6 independent measurements and SE are shown. The asterisks indicate significant differences between the WT and the OE line by one-way ANOVA with post-hoc Tukey HSD test ($p < 0.05$). Graphs were normalized to the initial point. (c) Effects of the inhibitors on cytosolic ATP concentration. Inhibitors of complex I (50 µM rotenone), complex II (100 µM TTFA) and complex V (10µM oligomycin) were applied to 10-day-old WT and OE7 seedling expressing C-AT1.03 seedlings by vacuum infiltration for 5 min. Before the measurements, the seedlings were incubated in the dark for 1 hour. Average value of 3 replicates is presented. Error bars are the standard error of the average $MgATP^{2-}$ concentration. Groups with significant difference by one-way ANOVA with post-hoc Tukey HSD test ($p < 0.05$) are indicated by different letters. The dashed line indicates the maximal $MgATP^{2-}$ concentration (1.4 mM) that can be reported by AT1.03 (Voon et al., 2018). The groups marked by asterisks are significantly different from the groups marked by letters but whether they have intergroup differences are not known.

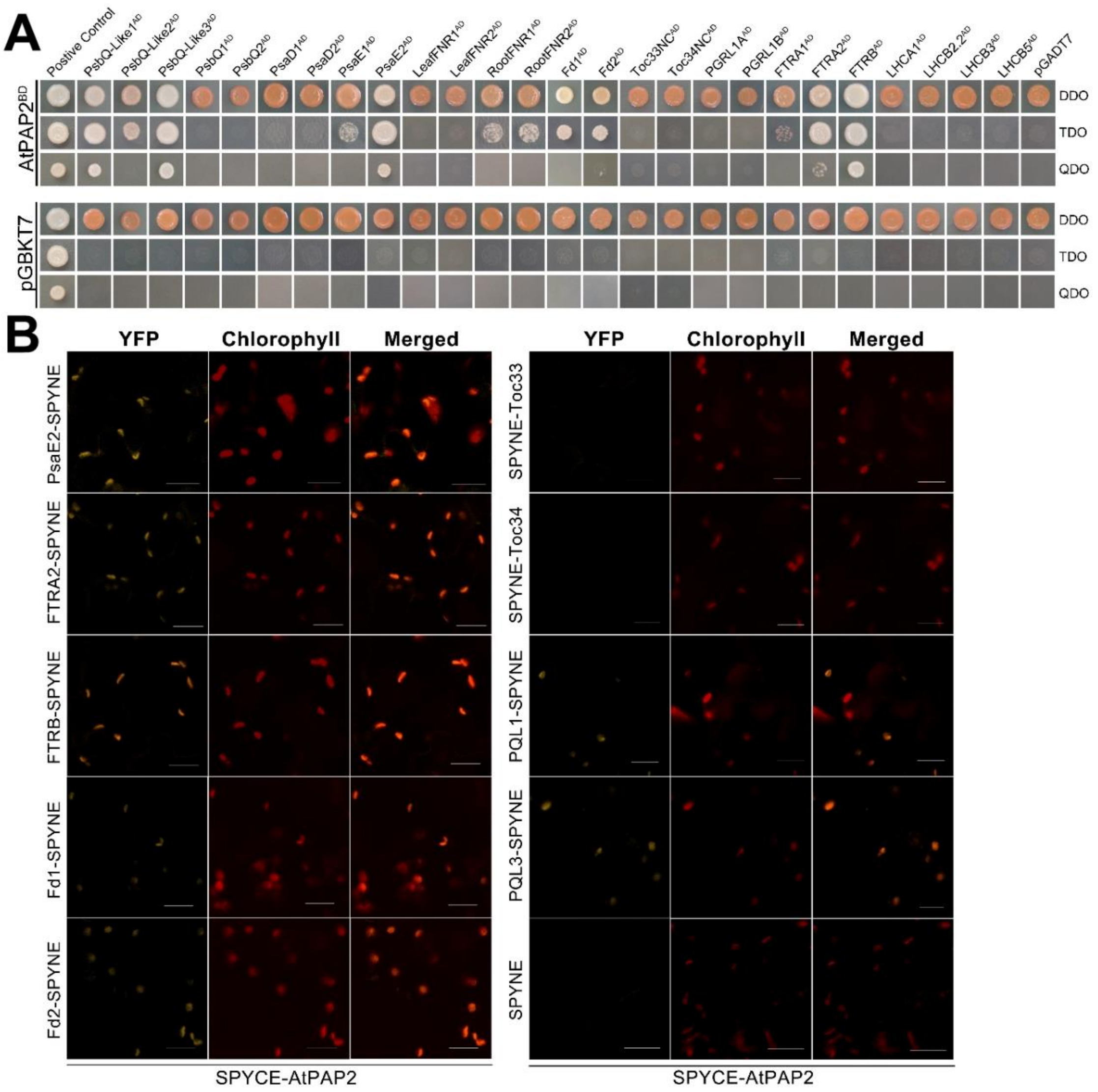

Supplementary Figure S7. AtPAP2 selectively interacts with certain photosystem proteins. A. Mature AtPAP2 protein without the signal peptide and C-terminus (a.a. 25–613, P2NC) interacted with several photosystem proteins (PQL1-AD, PQL2-AD, PQL3-AD, PsaE2-AD, FTRA2-AD and FTRB-AD). Blank vectors of BD (pGBKT7) and AD (pGADT7) were co-transformed with bait and prey, respectively, to assess auto-activation. The assays were carried on dropout medium of DDO (SD/-Leu/-Trp), TDO (SD/-Leu/-Trp/-His) and QDO (SD/-Ade/-His/-Leu/-Trp). **B**. BiFC assay of the interaction between AtPAP2 and candidate proteins. YFP^N-fused photosystem proteins (PsaE2, FTRA2, FTRB, Fd1, Fd2, PQL1, PQL3, Toc33 and Toc34) and YFP^C-AtPAP2 were transiently expressed in tobacco leaves. Empty SPYNE vectors were used as controls. Reconstituted YFP fluorescence was monitored at 514 nm (left panel with PMT detector emission bandwidth of 488–550 nm). Chlorophyll autofluorescence was excited at 458 nm (middle panel with PMT detector emission bandwidth of 650–710 nm). An overlay of the YFP signal and the chlorophyll autofluorescence is shown in the right panel. Scale bars: 20 µm. All images were captured using the same gain settings as the corresponding PMT channels. The representative images of 3 biological replicates are presented.

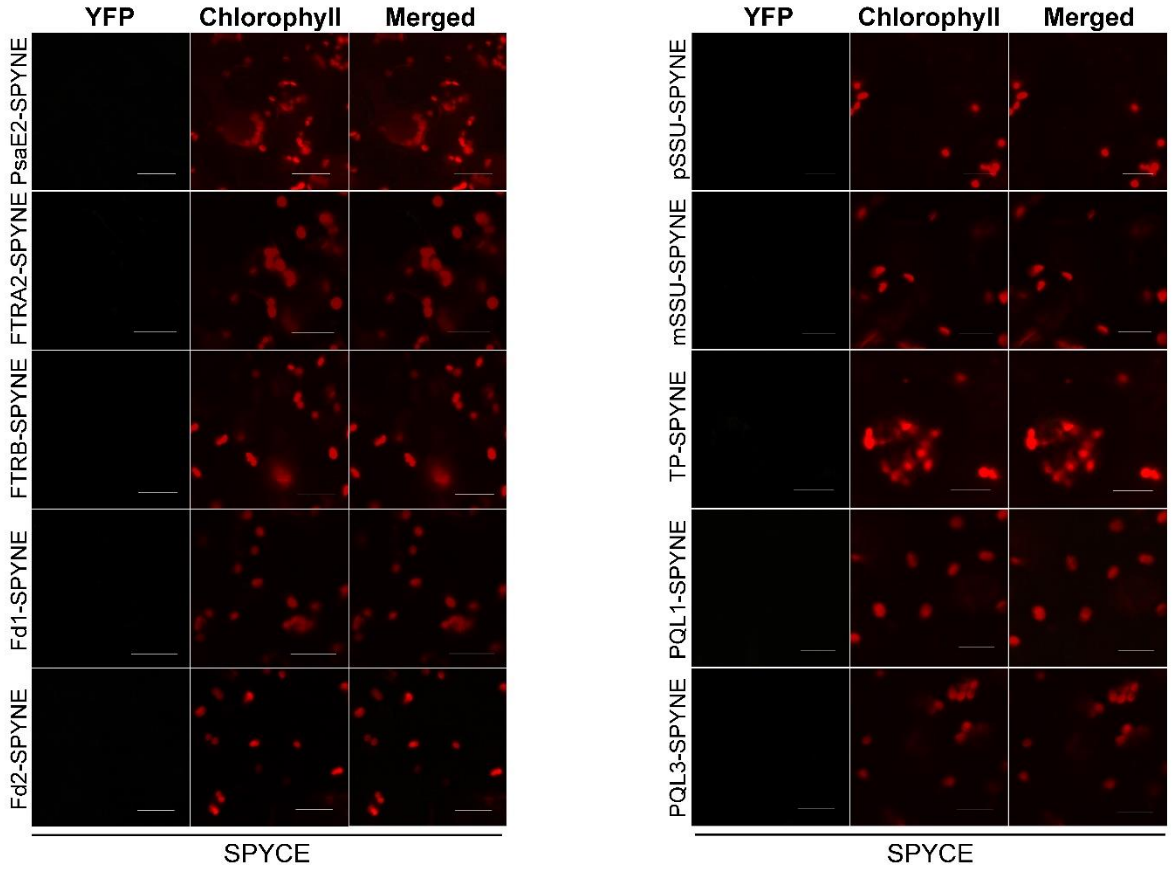

Supplementary Figure S8. Results of BiFC negative controls. YFPN-fused photosynthetic apparatus subunits (PsaE2, FTRA2, FTRB, PQL1, PQL3, Fd1, Fd2, SP, mSSU and pSSU) and empty SPYCE vector were transiently expressed in tobacco leaves as controls. Reconstituted YFP fluorescence was monitored at 514 nm (left panel with PMT detector emission bandwidth of 488–550 nm). Chlorophyll autofluorescence was excited at 458 nm (middle panel with PMT detector emission bandwidth of 650–710 nm). An overlay of the YFP signal and the chlorophyll autofluorescence is shown in the right panel. Scale bars: 20 μm. All of the images were captured at the same gain settings as the corresponding PMT channels.

Supplementary Table S1. Photosynthetic pigment content.

	WT	*pap2*	OE7	OE21
Chlorophyll a	8.43 + 0.44 [a]	10.62 + 0.18 [b]	6.53 + 1.12 [c]	5.55 + 0.29 [c]
Chlorophyll b	3.35 + 0.21 [a]	3.96 + 0.87 [b]	2.81 + 0.36 [ac]	2.16 + 0.16 [c]
β−Carotene	0.28 + 0.02 [a]	0.30 + 0.07 [b]	0.22 + 0.03 [c]	0.18 + 0.00 [ac]
Lutein	1.09 + 0.06 [a]	1.28 + 0.07 [b]	0.83 + 0.15 [c]	0.68 + 0.02 [c]
Violaxanthin	0.28 + 0.02 [a]	0.29 + 0.08 [a]	0.26 + 0.07 [a]	0.18 + 0.01 [a]

Pigments were extracted 8 hours after the light period from the leaves of 20-day-old Arabidopsis under LD regime. Values (mg g^{-1} DW of tissues) marked by different letters are significantly different ($p < 0.05$) in the same row by one-way ANOVA analysis followed by HSD test. DW, dry weight.

Supplementary Table S2. Chloroplast proteins identified in 2D BN PAGE.

Spot ID	Peptides (95%)	Unused peptide	Protein Name	Accession	Function	Average Ratio (OE/WT)
1	103	162.86	PsaA	ATCG00350	PS I core protein	1.00
1	80	98.48	PsaB	ATCG00340	PS I core protein	1.00
2	38	69.11	PsaA	ATCG00350	PS I core protein	0.82
2	38	62.91	PsaB	ATCG00340	PS I core protein	0.82
23	72	78.56	PsaD1	AT4G02770	PS I core protein	0.97
12	23	29.77	PsbA, D1	ATCG00020	PS II core protein	0.78*
6	95	82.02	PsbB, CP47	ATCG00680	PS II core protein	0.63**
7	58	96.16	PsbB, CP47	ATCG00680	PS II core protein	0.72*
8	51	64.74	PsbC, CP43	ATCG00680	PS II core protein	0.65**
9	181	249.7	PsbC, CP43	ATCG00280	PS II core protein	0.80
12	41	58.68	PsbD, D2	ATCG00270	PS II core protein	0.78*
13	108	136.85	PsbO1, OE33	AT5G66570	PS II core protein	6.83**
14	59	83.54	PsbO1, OE33	AT5G66570	PS II core protein	8.64**
15	15	26.96	PsbO1, OE33	AT5G66570	PS II core protein	3.02**
21	22	25.47	PsbS, NPQ4	AT1G44575	PS II core protein	1.06
22	25	39.68	PsbS, NPQ4	AT1G44575	PS II core protein	1.01
16	145	173.1	Lhcb1.4	AT2G34430	LHC II protein	0.88
16	57	27.88	Lhcb2.2	AT2G05070	LHC II protein	0.88
17	91	140.01	Lhcb4.1, CP29	AT5G01530	LHC II protein	1.44
18	59	63.18	Lhcb4.1, CP29	AT5G01530	LHC II protein	0.79
18	33	33.7	Lhcb1.4	AT2G34430	LHC II protein	0.79
19	92	10.33	Lhcb1.1	AT1G29920	LHC II protein	0.92
19	93	129.87	Lhcb1.4	AT2G34430	LHC II protein	0.92
19	47	33.61	Lhcb2.2	AT2G05070	LHC II protein	0.92
19	37	52.97	Lhcb5, CP26	AT4G10340	LHC II protein	0.92
20	129	12.78	Lhcb1.3	AT1G29930	LHC II protein	0.84
20	125	190.01	Lhcb1.4	AT2G34430	LHC II protein	0.84
20	57	47.46	Lhcb2.2	AT2G05070	LHC II protein	0.84
20	56	92.39	Lhcb5, CP26	AT4G10340	LHC II protein	0.84
21	29	35.92	Lhcb6, CP24	AT1G15820	LHC II protein	1.06
22	71	99.62	Lhcb6, CP24	AT1G15820	LHC II protein	1.01
24	32	45.98	PetC	AT4G03280	Cytochrome B6f complex	1.1
25	3	3.36	PetD	ATCG00730	Cytochrome B6f complex	0.25**
4	306	320.72	atpA	ATCG00120	ATP synthase	0.32**
5	325	381.21	atpB	ATCG00480	ATP synthase	0.36**
10	64	87.68	atpC1	AT4G04640	ATP synthase	0.60**
11	58	62.97	atpC1	AT4G04640	ATP synthase	0.66**
3	271	405.15	RBCL	ATCG00490	Calvin cycle	4.07**

Isolated chloroplast proteins from WT and OE7 were resolved by 2D BN/SDS-PAGE and the protein spots were extracted for MS/MS analysis. Protein candidates with the highest unused score and at least 2 unique peptides (95%) were shown. Average ratio is the mean of the value (OE/WT) from three biological replicates. Spots with fold change of ±1.2 or ±1.5 folds and $p < 0.05$ by one-way ANOVA are denoted by * and **, respectively.

Supplementary Table S3. Summary of identified mitochondrial protein spots.

Spot ID	Peptides (95%)	Unused peptide	Protein name	Accession	Protein complex	Ratio (OE/WT)
1	327	328.85	Heat Shock Protein 60-3B	AT3G23990.1	HSP60	1.07
1	262	158.77	Heat Shock Protein 60-2	AT2G33210.1	HSP60	1.07
2	244	288.80	MPPbeta	AT3G02090.1	Complex III	0.99
3	295	358.38	ATP synthase beta-subunit	AT5G08690.1	Complex V	0.52**
3	292	4.48	ATP synthase beta-subunit	AT5G08680.1	Complex V	0.52**
3	239	312.37	ATP synthase alpha-subunit	AT2G07698.1	Complex V	0.52**
4	217	287.35	ATP synthase beta-subunit	AT5G08690.1	Complex V	0.43**
4	209	6.00	ATP synthase beta-subunit	AT5G08680.1	Complex V	0.43**
4	8	234.30	ATP synthase alpha-subunit	AT2G07698.1	Complex V	0.43**
5	622	637.08	RBCL	ATCG00490.1		2.33**
6	342	349.23	ATP synthase beta-subunit	AT5G08690.1	Complex V	0.56**
6	335	6.00	ATP synthase beta-subunit	AT5G08680.1	Complex V	0.56**
6	204	220.55	ATP synthase alpha-subunit	AT2G07698.1	Complex V	0.56**
7	245	315.84	Insulinase protein	AT1G51980.1	Complex III	1.08
7	73	11.84	MPPalpha	AT3G16480.1	Complex III	1.08
7	14	22.21	MPPbeta	AT3G02090.1	Complex III	1.08
8	242	335.95	ATP synthase gamma-subunit (ATP3)	AT2G33040.1	Complex V	0.64**
8	25	41.00	ATP Synthase subunit 1 (ATP1)	ATMG01190.1	Complex V	0.64**
8	20	35.36	ATP synthase beta-subunit	AT5G08690.1	Complex V	0.64**
9	19	28.61	ATP synthase alpha-subunit	AT2G07698.1	Complex V	0.65**
9	32	56.70	ATP synthase gamma-subunit (ATP3)	AT2G33040.1	Complex V	0.65**
9	29	50.43	ATP synthase beta-subunit	AT5G08690.1	Complex V	0.65**
10	87	131.85	ATP synthase gamma-subunit (ATP3)	AT2G33040.1	Complex V	0.64**
10	49	72.64	ATP synthase beta-subunit	AT5G08670.1	Complex V	0.64**

Isolated mitochondrial proteins from WT and OE7 were resolved by 2D BN/SDS-PAGE and the mitochondrial protein spots were extracted for MS/MS analysis. Protein candidates with the highest unused score and at least 2 unique peptides (95%) were shown. Average ratio is the mean

of the value (OE/WT) from three biological replicates. Spots with fold change of ±1.2 or ±1.5 folds and $p < 0.05$ by one-way ANOVA are denoted by * and **, respectively.

Supplementary Table S4. Respiration rates of different complexes of WT and OE7 mitochondria.

Enzyme	Substrate	ADP	Rotenone	WT	OE7	Change
Complex I	deamino-NADH	-	-	30 ± 6	33 ± 5	ns
		-	+	8 ± 3	8 ± 5	ns
Complex II	Succinate	-	-	49 ± 3	48 ± 23	ns
		+	-	75 ± 4	92 ± 10 *	1.23X
NdEx	NADH	-	-	45 ± 4	45 ± 12	ns
		+	-	65 ± 4	68 ± 4	ns
NdIn	Malate + Glutamate	-	+	39 ± 4	46 ± 6	ns
		+	+	64 ± 8	85 ± 4 *	1.32X

Oxygen consumption rates of complex I (rotenone-sensitive deamino-NADH oxidation), complex II, external NADH dehydrogenase, internal NADH dehydrogenase (rotenone-insensitive NADH oxidation) were expressed as nmol min^{-1} mg^{-1} protein.

* indicates statistically significant difference ($p < 0.05$, $n \geq 3$) and ns indicates not significant difference between WT and OE7 by Student's t test.

Supplementary Table S5. AtPAP2-interacting proteins identified by Y2H library screening.

AGI code	No. of clones	a.a. residues	Sequence in clone	Phosphorylation sites (1-50)	pI	Proteins	Experimental location
At1g14150	5	190	All are full length	T9,Y13,T46	8.93	PsbQ-like (PQL1)	Plastid
At1g18640	5	695	All are full length	*T6*	6.29	3-Phosphoserine phosphatase	Plastid
At1g26220	1	197	Full length	T9,T27,S31,Y46,S47,S49	9.79	Acyl-CoA N-acyltransferases superfamily protein	Plastid
At1g29900	2	1187	aa.771-End	*S18,S19,S26,S31,S33*	5.45	Carbamoyl phosphate synthetase B (CARB)	Plastid
At1g30510	1	382	aa.135-End	S15,T49	8.85	Ferredoxin--NADP+ reductase (RFNR2)	Plastid
At2g04700	5	146	All are full length	S15,T20,T34,S48	7.98	Ferredoxin-thioredoxin reductase B	Plastid
At2g05990	1	390	aa.149-End	S17,S19,S20,Y30	9.36	Enoyl-ACP reductase	Plastid
At2g20260	1	145	Full length	S24,S25	10.54	Subunit E of photosystem I (PsaE2)	Plastid
At2g22360	1	442	aa.228-End	Y25,S32	9.49	DNAJ heat shock family protein A6	Plastid
At2g34860	1	186	Full length	S16,S39,S42,S45,S47,S49	9.25	DnaJ/Hsp40 cysteine-rich domain protein	Plastid
At2g35500	1	387	Full length	*S14,S34,S35*	6.19	Shikimate kinase-like 2 (SKL2)	Plastid
At4g20760	1	298	Full length	S14,S34,S37,T32	10.24	NAD(P)-binding Rossmann-fold protein	Plastid
At4g21860	1	202	Full length	T7,S34,S45,S46	8.68	Methionine sulfoxide reductase B2	Plastid
At4g25370	1	238	Full length	S26,S28,S29,T36	9.78	Double Clp-N motif protein	Plastid
At4g35860	1	211	Full length	T49	7.03	GTP-binding protein GB2 (GB2)	Plastid
At5g06340	3	227	All are full length	S37,S50	8.77	Nudix hydrolase 27 (NUDX27)	Plastid
At5g08280	1	382	Full length	*T26,S44*	8.62	Hydroxymethylbilane synthase	Plastid
At5g11450	4	297	All are full length	S20,Y27,T43,S45,S47	9.06	PPD5	Plastid

At5g11650	1	390	aa.323-End	*T12*	8.73	Alpha/beta fold hydrolase family protein	Plastid
At5g19370	4	299	All are full length	T7,S9,S13,T30	8.62	Rhodanese-like Peptidyl-prolyl cis-trans isomerase	Plastid
At5g21222	1	831	aa.523-End	Y13,T33,T35	8.01	Serine/threonine protein kinase	Plastid
At5g38430	2	181	Full length	S14,S32,T36	7.83	Ribulose bisphosphate carboxylase small chain 1B	Plastid
At5g66120	2	442	aa.1-344 and 1-343	T14,T31,Y50	7.53	3-dehydroquinate synthase	Plastid
At4g33520	1	949	Full length	T14	9.02	Copper-transporting ATPase PAA1 (PAA1)	Plastid inner membrane
At1g53670	1	202	Full length	Y23,Y48	9.20	Methionine sulfoxide reductase B1	Plastid stroma
At2g20270	1	206	Full length	T45	8.94	Monothiol glutaredoxin-S12	Plastid stroma
At2g38270	4	293	All are full length	S15,T31,T37,S41	8.07	Monothiol glutaredoxin 16	Plastid stroma
At2g36460	2	358	aa.76-End and aa.112-End	S32,T33,T35,S42	7.46	Fructose-bisphosphate aldolase, class I	Plastid, cytosol, PM, extracellular
At1g18080	1	327	aa.53-End	T19,T23	7.81	RACK1A	Plastid, cytosol, PM, nucleus
At1g70580	1	481	aa.145-End	Y8,Y27	6.52	Alanine-2-oxoglutarate aminotransferase 2	Plastid, Peroxisome
At5g16370	1	552	aa. 871-1766	S11,T15	6.97	Acyl activating enzyme 5 (AAE5)	Plastid, Peroxisome
At4g25130	3	258	All are full length	*S7*	8.92	Methionine sulfoxide reductase	Plastid, stroma
At2g35240	1	232	Full length	S19,S26,S40,Y49	9.14	pMORF6	Mitochondria
At5g40770	1	277	aa.47-End	T34	7.89	Prohibitin 3 (PHB3)	Mitochondria
At4g35830	1	898	aa.467-End	Y41	6.35	Aconitate hydratase 1 (ACO1)	Mitochondria and cytosol
At1g05060	1	253	Full length	Y16,S32,T35,S41,S42	10.02	Uncharacterized protein	N/A
At1g29810	1	187	Full length	T42,S47,T49,T50	10.37	Transcriptional coactivator/pterin dehydratase	N/A

At1g31817	1	314	aa.48-End	S31,S33,S37,S48	11.15	30S ribosomal protein S11	N/A
At1g53140	1	817	aa.620-End	Y7,T10,T14	7.47	Dynamin related protein 5A (DRP5A)	N/A
At2g38080	1	558	aa.172-534	T49	9.60	Laccase/Diphenol oxidase family protein	N/A

Experimental location: determined by GFP or MS analysis (SUBA3, http://suba.plantenergy.uwa.edu.au/)
Phosphorylation sites (1-50): Experimental determined phophorylation sites in the first 50 a.a. residues were retrieved from Phosphat 4.0 (http://phosphat.uni-hohenheim.de/phosphat.html)
Predicted location: predicted by SUBA3 (http://suba.plantenergy.uwa.edu.au/)
Predicted phosphorylation sites (1-50) are shown in italics. The prediction was carried out at Phosphat 4.0 or NetPhos 2.0 (http://www.cbs.dtu.dk/services/NetPhos/)

Supplementary Table S6. Primer list.

Vector (Purpose)	Primer Name (AGI code)	Primer Sequence (5’-3’)[1]
pGBKT7	25-613aaAtPAP2	F: TTCTCATATGACCATTTCAATTTCCCC
(Yeast two-hybrid)	(AT1G13900)	R: GCATGTCGACCAGCATTAGATTCTGATTTTC
pGADT7	42-181aaSSU1B	F: ATTCCATATGGACATTACTTCCATCACAAGC
(Yeast two-hybrid)	(AT5G38430)	R: ATTACTCGAGAGCATCAGTGAAGCTTGGGG
pGADT7	1-50aaSSU1B	F: ATTACATATGGCTTCCTCTATGCTCTCCT
(Yeast two-hybrid)	(AT5G38430)	R: ATTACTCGAGCCCATTGCTTGTGATGGAAG
pGADT7	PsbQ1	F: ATTAGAATTCATGGCTTCGATGGGTGGATT
(Yeast two-hybrid)	(AT4G21280)	R: CTAGCTCGAGTTAACCAAGCTTGGCAAGAACT
pGADT7	PsbQ2	F: TTAGCATATGATGGCTCAAGCAGTGACTTC
(Yeast two-hybrid)	(AT4G05180)	R: ATTAGAGCTCTTAACCGAGCTTGGCAAGAAC
pGADT7	PsbQ-Like1	F: ATTGCATATGATGAGCTCCTTCACCACCAC
(Yeast two-hybrid)	(AT1G14150)	R: ATCGCTCGAGTTAAGCAAGAAACTCCACAAC
pGADT7	PsbQ-Like2	F: TGTCCATATGATGGCTCACTTCATAGACCT
(Yeast two-hybrid)	(AT3G01440)	R: TAATCTCGAGTCATGCCATTCTGGTCATTAC
pGADT7	PsbQ-Like3	F: TAATCATATGATGGCGATTTCAAAGCCACC
(Yeast two-hybrid)	(AT2G01918)	R: ACCGCTCGAGTTAAATTCGAGGGAAGATATC
pGADT7	PsaD1	F: ATTACATATGATGGCAACTCAAGCCGCCGG
(Yeast two-hybrid)	(AT4G02770)	R: GCTACTCGAGTTACAAATCATAACTTTGTTTGCCAG
pGADT7	PsaD2	F: ATTACATATGATGGCAACTCAAGCCGCCGG
(Yeast two-hybrid)	(AT1G03130)	R: GTCTCGAGTTACAAATCATAAGATTGTTTCCCAGTG
pGADT7	PsaE1	F: ATTACATATGATGGCGATGACGACAGCATC
(Yeast two-hybrid)	(AT4G02770)	R: CTTACTCGAGTTAAGCTGCAACTTCTTCGACCT
pGADT7	PsaE2	F: ATTACATATGATGGCGATGACGTCAGCAGC
(Yeast two-hybrid)	(AT2G20260)	R: GTTACTCGAGTCATTTTACTTCTTCCACCTCGTCC
pGADT7	LeafFNR1	F: TTAGCATATGATGGCTGCTGCTATAAGTGC
(Yeast two-hybrid)	(AT5G66190)	R: TACGCTCGAGTTAGTAGACTTCAACATTCCACTG
pGADT7	LeafFNR2	F: ATTACATATGATGGCGACTACCATGAATGC
(Yeast two-hybrid)	(AT1G20020)	R: TCTACTCGAGTCAGTAGACTTCAACGTTCCATT
pGADT7	RootFNR1	F: ATTGCATATGATGGCTCTCTCAACTACTCC
(Yeast two-hybrid)	(AT3G05390)	R: GCTACTCGAGTCAATACACTTCAACATGCC
pGADT7	RootFNR2	F: ATTGCATATGATGTCTCACTCTGCTGTTTCT
(Yeast two-hybrid)	(AT1G30510)	R: CTTACTCGAGTCAATAGACTTCAACGTGCC
pGADT7	LeafFd1	F: ATTACATATGATGGCTTCCACTGCTCTCTCC
(Yeast two-hybrid)	(AT1G10960)	R: ATTACTCGAGTTACATAATGGCTTCTTCTTTGTGG
pGADT7	LeafFd2	F: ATTGCATATGATGGCTTCCACTGCTCTCTC
(Yeast two-hybrid)	(AT1G60950)	R: GTCACTCGAGTTAAACAATGTCTTCTTCTTTGTGG
pGADT7	1-251aaToc33	F: ATTACATATGATGGGGTCTCTCGTTCGTG
(Yeast two-hybrid)	(AT1G02280)	R: ATTACCCGGGTTAGTCTACATGAATTGCTTTCCTC
pGADT7	1-254aaToc34	F: ATTACATATGATGGCAGCTTTGCAAACGCT
(Yeast two-hybrid)	(AT5G05000)	R: ATTACCCGGGTTACTTGTCAACATGAATCGCCTT
pGADT7	PGR5-Like1A	F: ATTGCATATGATGGGTAGCAAGATGTTGTT
(Yeast two-hybrid)	(AT4G22890)	R: TAATCTCGAGTTAAGCTTGGCTTCCTTCTG
pGADT7	PGR5-Like1B	F: ATTGCATATGATGGCTTTTACTCTAACAATCC
(Yeast two-hybrid)	(AT4G11960)	R: AACTGAGCTCTTAAGCTTTCCCTCCTTCTG
pGADT7	FTRA1	F: ATTGCATATGATGAGTAGCCAAATCGCTTTGT
(Yeast two-hybrid)	(AT5G23440)	R: TACTGAGCTCTCACTGATCAATGAACTCGAACTC
pGADT7	FTRA2	F: ATTGCATATGATGACTAACAGTTACGCTCTGTC
(Yeast two-hybrid)	(AT5G08410)	R: ATTACTCGAGTCACGGATCAATTAACTCGAAC
pGADT7	FTRB	F: AGCTGAATTCATGAATCTTCAAGCTGTTTC
(Yeast two-hybrid)	(AT2G04700)	R: ATCGCTCGAGTCACATGTTAGCTGTAGTTTC

pGADT7	LHCA1	F: TAATCATATGGCGTCGAACTCGCTTAT
(Yeast two-hybrid)	(AT3G54890)	R: TAATCATATGTTAGTTGAAAGGGATAACAAT
pGADT7	LHCB2.2	F: TAATCATATGGCCACATCAGCTATCCAAC
(Yeast two-hybrid)	(AT2G05070)	R: TAATCTCGAGTTACTTTCCGGGGACAAAGTT
pGADT7	LHCB3	F: TAATCATATGGCATCAACATTCACGAGC
(Yeast two-hybrid)	(AT5G54270)	R: TAATCTCGAGTTAAGCTCCAGGTGCAAACT
pGADT7	LHCB5	F: TAATCATATGGCGTCTTTGGGTGTGTC
(Yeast two-hybrid)	(AT4G10340)	R: TAATCTCGAGTTAGAGAGTGGGAGCTCTCTC
SPYCE	AtPAP2	F: ATCGACTAGTATGATCGTTAATTTCTCTTTCTTC
(BiFC)	(AT1G13900)	R: GAATACTAGTTTATGTCTCCTCGTTCTTGACTG
SPYCE	Multiple cloning site	F: ATTAGAGCTCGTTAACCGGGCTCAGGCCT
(BiFC)		R: ATTAGAGCTCCCCGGGAGCGGTACCCTC
SPYCE	YFP^C	F: TAACTCTAGAATGTACCCATACGATGTTCCAG
(BiFC)		R: ATTAGAGCTCCTTGTACAGCTCGTCCATG
SPYNE	PsaE1	F: GAACTCTAGAATGGCGATGACGACAGCAT
(BiFC)	(AT4G28750)	R: ATTACTCGAGAGCTGCAACTTCTTCGACCTC
SPYNE	PsaE2	F: ATTATCTAGAATGGCGATGACGTCAGCAGC
(BiFC)	(AT2G20260)	R: TAATCTCGAGTTTTACTTCTTCCACCTCGTCCAAT
SPYNE	FTRA1	F: ATTATCTAGAATGAGTAGCCAAATCGCTTT
(BiFC)	(AT5G23440)	R: ATTACTCGAGCTGATCAATGAACTCGAACTC
SPYNE	FTRA2	F: CTAGTCTAGAATGACTAACAGTTACGCTCTGTC
(BiFC)	(AT5G08410)	R: ATTACTCGAGCGGATCAATTAACTCGAACTC
SPYNE	FTRB	F: ATTATCTAGAATGAATCTTCAAGCTGTTTCTTG
(BiFC)	(AT2G04700)	R: ATTACTCGAGCATGTTAGCTGTAGTTTCTTTTATT
SPYNE	LeafFd1	F: ATTATCTAGAATGGCTTCCACTGCTCTCTCC
(BiFC)	(AT1G10960)	R: TAATCTCGAGCATAATGGCTTCTTCTTTGTGG
SPYNE	LeafFd2	F: ATTGTCTAGAATGGCTTCCACTGCTCTCTC
(BiFC)	(AT1G60950)	R: ATTACTCGAGAACAATGTCTTCTTCTTTGTGGG
SPYNE	Toc33	F: ATTATCTAGAATGGGGTCTCTCGTTCGTGAAT
(BiFC)	(AT1G02280)	R: CCTATCTAGAAAGTGGCTTTCCACTTGTCTTGAT
SPYNE	Toc34	F: ATTATCTAGAATGGCAGCTTTGCAAACGCT
(BiFC)	(AT5G05000)	R: GAATTCTAGAAGACCTTCGACTTGCTAAACCG
SPYNE	PsbQ-Like1	F: ATTGACTAGTATGAGCTCCTTCACCACCAC
(BiFC)	(AT1G14150)	R: ATTACTCGAGAGCAAGAAACTCCACAACATT
SPYNE	PsbQ-Like3	F: ATTCACTAGTATGGCGATTTCAAAGCCACC
(BiFC)	(AT2G01918)	R: ATTACTCGAGAATTCGAGGGAAGATATCATCGAG
SPYNE	42-181aaSSU1B	F: ATTCTCTAGAATGGACATTACTTCCATCACAAGC
(BiFC)	(AT5G38430)	R: ATTACTCGAGAGCATCAGTGAAGCTTGGGG
SPYNE	1-50aaSSU1B	F: ATTATCTAGAATGGCTTCCTCTATGCTCTCCT
(BiFC)	(AT5G38430)	R: ATTACTCGAGCCCATTGCTTGTGATGGAAG
pCXSN	TOC33overlap	F: ATCAGTGGGAGCAAAGTAGATGGATCTTACTCT
(Transgenic line)	(At1g02280)	R: GTAAGATCCATCTACTTTGCTCCCACTGATTAC

[1] Restricted sites are indicated by underline.

Supplementary Methods

Determination of the chlorophyll content using an HPLC analysis. Leaves (30-50 mg) were freeze-dried and extracted 3 times using 100% acetone (500 μl, 250 μl and 250 μl). The mixture was combined and centrifuged twice to eliminate the insoluble substances prior to the HPLC analysis. The pigment extract was separated and analysed (20 μL aliquots) on a Waters Spherisorb 5 μm ODS2 (4.6 × 250 mm) analytical column (Waters, USA) at room temperature. The fluids were eluted at a flow rate of 1.2 ml min^{-1} with a linear gradient from 100% solvent A [acetonitrile/methanol/0.1 M TRIS–HCl (pH 8.0), 84:2:14, v/v/v] to 100% solvent B (methanol/ethyl acetate, 68:32, v/v) over a 15-min period, followed by 10 min of 100% solvent B. The tridimensional chromatogram was recorded from 250 to 700 nm. The chromatographic peaks were identified by comparing the retention times and spectra to known standards provided by Sigma (USA) and Wako (Japan). The pigments were finally quantified by integrating the peak areas and converting them to concentrations according to the comparisons. The amounts of lutein, β−carotene, violaxanthin, chlorophyll a (Ca) and chlorophyll b (Cb) were calculated as follows: lutein: $Y = 2.56 \times 10^8 X - 153665$, $R = 0.99577$, $p < 0.0001$; β-carotene: $Y = 6.57 \times 10^8 X – 61818$; violaxanthin: $Y = 2.68 \times 10^8 X + 236.6$; Chl a: $Y = 3.30 \times 10^7 X + 38832$; and Chl b: $Y = 7.31 \times 10^7 X + 29646$. Y = area, X = mg/ml.

Transmission electron microscopy (TEM). Plant materials (5th to 7th true leaves of 19-day-old plants at the beginning of the light period) were fixed with 2.5% (v/v) glutaraldehyde in 75 mM sodium cacodylate and 2 mM $MgCl_2$, pH 7.0, for 1 hour at 25 °C and post-fixed for 2 hours with 1% (w/v) osmium tetroxide in a fixative buffer at 25 °C. After two washing steps with distilled water, the pieces were dehydrated and then embedded in Spurr's low-viscosity resin. Ultrathin sections of 50–70 nm were cut with a diamond knife. The sections were post-stained with aqueous uranyl acetate/lead citrate. The TEM examination was performed under a Hitachi H-7650 transmission electron microscope with a charge-coupled device camera (Hitachi High-Technologies, Japan) operating at 80 kV.

2D Blue Native PAGE DIGE. Leaves from 20-day-old soil-grown WT and OE7 plants at the middle of the day (T = 8) were used as the starting materials. The chloroplast (Lamkemeyer et al., 2006) and mitochondria isolation (Lee et al., 2008) were performed as previously described. Cy

Dye labelling of the chloroplast or mitochondrial proteins was carried out as previously described with modifications (Heinemeyer et al., 2009). In total, 100 µg chloroplast/mitochondrial proteins were centrifuged for 10 min at 4 °C at 1000 g using an Eppendorf centrifuge. The sedimented chloroplasts or mitochondria were re-suspended in a 10 µl solubilization buffer containing 30 mM HEPES, pH 7.4, 150 mM potassium acetate, 10% [v/v] glycerol, supplemented with 2% [w/v] beta-dodecyl maltoside (chloroplasts) or 5% [w/v] digitonin (mitochondria). The solubilized proteins were centrifuged for 20 min at 4 °C at full speed to remove the insoluble material. The supernatant containing the solubilized protein complexes was supplemented with 10 µl of the solubilization buffer containing 30 mM HEPES, pH 10, 150 mM potassium acetate, 10% [v/v] glycerol to adjust the pH value of the protein solution to approximately 8.5, which is a prerequisite for efficient labelling. Cy2, Cy3 and Cy5 NHS ester minimal dyes (Lumiprobe Corporation, USA) were reconstituted into 400 pmol/µl in DMF according to the manufacturer's instructions, and 2 µl of the Cy dye were added to the solubilized chloroplast or mitochondrial proteins. The WT was labelled using a Cy3 dye, and the OE was labelled with the Cy5 dye, while the internal standard (consisting of a pooled sample comprising an equal amount of all samples in the experiment) was labelled with Cy2. The labelling occurred for 30 min in the dark on ice and was stopped by adding 1 µl of a Lysine solution (10 mM). Finally, the sample was supplemented with 3 µl 5% (w/v) Serva Blue G (750 mM aminocaproic acid, 5% [w/v] Coomassie 250 G) and directly transferred into the slot of a blue native gel. The solubilized proteins were separated by two-dimensional Blue native/SDS PAGE as described previously by Wittig et al. (2006). The image acquisition was performed with a Typhoon Scanner, and the quantification of the Cy Dye labelled proteins resolved by the 2D Blue native PAGE was carried out using the SAMESPOTS software (Totallab, Tyne, England) based on the gel-images acquired from 3 biological replicates.

Ruptured chloroplast assay. The cyclic electron flow was measured in ruptured chloroplasts through an Fd-dependent plastoquinone reduction as previously described (Endo et al., 1998; Munekage et al., 2002). Briefly, the isolated chloroplasts (10 µg Chl) were osmotically ruptured in the analysis buffer (7 mM $MgCl_2$, 1 mM $MnCl_2$, 2 mM EDTA, 30 mM KCl, 0.25 mM KH_2PO_4, and 50 mM HEPES, pH 7.6) for 10 min prior to the analysis. One min after the initiation of the measurement, NADPH (Roche, Basel, Switzerland) was added to a final concentration of 0.25 mM. Maize ferredoxin (Sigma Aldrich, St. Louis, MO, USA) was added after an additional 30s.

FRET-based ATP sensor and pH-dependent fluorescent probe. The use of ATP sensor AT1.03 in plant systems was recently verified (Voon et al., 2018). A pH-dependent fluorescent probe with circularly permuted yellow fluorescent protein (cpYFP) was introduced into the matrix of the mitochondria (Nagai et al., 2001). Protonated cpYFP displays a higher absorption at 395 nm, which emits a higher signal at 528 nm if excited at 395 nm. cpYFP was shown to response specifically to the pH value (Schwarzländer et al., 2011).

Bimolecular fluorescence complementation (BiFC). The full-length coding sequence of the bait protein AtPAP2 was fused to the C-terminus of YFPC in the pSPYCE vector, while the coding sequences of the photosynthetic apparatus subunits were fused to the N-terminus of the pSPYNE containing YFPN (Kerppola, 2006). Both the prey and bait plasmids were transformed into the Agrobacterium tumefaciens strain GV3101, and the bacteria were infiltrated into the epidermal cell layers of tobacco leaves as previously described (Schweiger and Schwenkert, 2014). The transfected regions of the leaves were used for fluorescence detection after a 48-hour incubation in the dark under an LSM710 confocal laser scanning microscope (Zeiss). The primers that were used in this study are listed in Supplemental Table S5.

Yeast two-hybrid assay. A normalized yeast two-hybrid (Y2H) library was prepared from mRNAs that were isolated from eleven *Arabidopsis* tissues and was employed for the screening (Clontech Laboratories, Japan). The coding sequence of the mature bait protein AtPAP2 (25-613 a.a.), which lacks its signal peptide and C-terminal transmembrane motif, was fused to the C-terminus of the GAL4 DNA-binding domain (BD) in the pGBKT7 vector. The GAL4-based Y2H library screening was performed using the Matchmaker Gold Yeast Two-Hybrid System following the manufacturer's instructions (Clontech Laboratories, Japan). The mated culture was concentrated by centrifugation and spread onto agar plates (90 mm) containing triple dropout medium without Leu, Trp and His (TDO). Putative positive colonies (> 2 mm) were cultured on TDO liquid medium, and pGADT7 plasmids were extracted from the yeast cells for the *E. coli* transformation. The plasmids (pGADT7-prey) were re-extracted and sequenced. To identify the interactions between AtPAP2 and various components of the photosynthetic apparatus, the coding sequences of prey proteins were amplified from *Arabidopsis* leaf cDNA by Platinum^{TM} *Pfx* polymerase (Thermo Scientific, USA) and fused downstream of the DNA-activating domain (AD) in the pGADT7 vector. The Y2HGold yeast cells were co-transformed with both the bait and prey

protein constructs using the lithium acetate method and plated onto double dropout without Leu and Trp (DDO), TDO, and quadruple dropout without Ade, His, Leu and Trp (QDO) agar plates (Clontech Laboratories, Japan).

References

Endo, T., Shikanai, T., Sato, F. and Asada, K. (1998) NAD(P)H dehydrogenase-dependent, antimycin A-sensitive electron donation to plastoquinone in tobacco chloroplasts. *Plant and Cell Physiology* **39**, 1226-1231.

Heinemeyer, J., Scheibe, B., Schmitz, U.K. and Braun, H.P. (2009) Blue native DIGE as a tool for comparative analyses of protein complexes. *J Proteomics* **72**, 539-544.

Kerppola, T.K. (2006) Design and implementation of bimolecular fluorescence complementation (BiFC) assays for the visualization of protein interactions in living cells. *Nat Protoc* **1**, 1278-1286.

Lamkemeyer, P., Laxa, M., Collin, V., Li, W., Finkemeier, I., Schottler, M.A., Holtkamp, V., Tognetti, V.B., Issakidis-Bourguet, E., Kandlbinder, A., Weis, E., Miginiac-Maslow, M. and Dietz, K.J. (2006) Peroxiredoxin Q of *Arabidopsis thaliana* is attached to the thylakoids and functions in context of photosynthesis. *Plant J* **45**, 968-981.

Lee, C.P., Eubel, H., O'Toole, N. and Millar, A.H. (2008) Heterogeneity of the mitochondrial proteome for photosynthetic and non-photosynthetic Arabidopsis metabolism. *Molecular & cellular proteomics : MCP* **7**, 1297-1316.

Munekage, Y., Hojo, M., Meurer, J., Endo, T., Tasaka, M. and Shikanai, T. (2002) PGR5 is involved in cyclic electron flow around photosystem I and is essential for photoprotection in Arabidopsis. *Cell* **110**, 361-371.

Nagai, T., Sawano, A., Park, E.S. and Miyawaki, A. (2001) Circularly permuted green fluorescent proteins engineered to sense Ca^{2+}. *Proc Natl Acad Sci U S A* **98**, 3197-3202.

Schwarzländer, M., Logan, D.C., Fricker, M.D. and Sweetlove, L.J. (2011) The circularly permuted yellow fluorescent protein cpYFP that has been used as a superoxide probe is highly responsive to pH but not superoxide in mitochondria: implications for the existence of superoxide 'flashes'. *Biochem J* **437**, 381-387.

Schweiger, R. and Schwenkert, S. (2014) Protein-protein Interactions visualized by bimolecular fluorescence complementation in tobacco protoplasts and leaves. *Jove-J Vis Exp* **85**, e51327.

Voon, C.P., Guan, X., Sun, Y., Sahu, A., Chan, M.N., Gardeström, P., Wagner, S., Fuchs, P., Nietzel, T., Versaw, W.K., Schwarzländer, M. and Lim, B.L. (2018) ATP compartmentation in plastids and cytosol of *Arabidopsis thaliana* revealed by fluorescent protein sensing. *Proc Natl Acad Sci U S A* **115**, 10778-10787.

Wittig, I., Braun, H.P. and Schagger, H. (2006) Blue native PAGE. *Nat Protoc* **1**, 418-428.
